# Evaluating the seasonality of growth in infants using a mobile phone application

**DOI:** 10.1038/s41746-020-00345-9

**Published:** 2020-10-21

**Authors:** Satoshi Narumi, Tetsu Ohnuma, Kenji Takehara, Naho Morisaki, Kevin Y. Urayama, Tomoyuki Hattori

**Affiliations:** 1 Department of Molecular Endocrinology, National Research Institute for Child Health and Development, Tokyo, 157-8535 Japan; 2 CAPER Unit, Department of Anesthesiology, Duke University School of Medicine, Durham, NC 27710 USA; 3 Department of Health Policy, National Center for Child Health and Development, Tokyo, 157-8535 Japan; 4 Department of Social Medicine, National Center for Child Health and Development, Tokyo, 157-8535 Japan; 5 Graduate School of Public Health, St. Luke’s International University, Tokyo, 104-0045 Japan; 6 First Ascent Inc., Tokyo, 104-0061 Japan

**Keywords:** Paediatric research, Paediatric research

## Abstract

It has been observed that growth velocity of toddlers and school children shows seasonal variation, while such seasonality is unknown in infants. The aim of this study was to examine whether growth velocity (length and weight) of infants differs by seasons. We assessed longitudinal measurement data obtained for 9,409 Japanese infants whose parents used the mobile phone application, “Papatto Ikuji”, during the period from January 2014 to October 2017. On average, each infant had 4.8 entries for length and 5.4 entries for weight. The mean daily change in sex- and age-adjusted *z-*scores between two time points was estimated as the growth velocity during that period: ΔLAZ/day and ΔWAZ/day for length and weight, respectively. We analyzed 20,007 ΔLAZ/day (mean, −0.0022) and 33,236 ΔWAZ/day (mean, 0.0005) measurements, and found that ΔLAZ/day showed seasonal differences with increases during summer. We conducted a multilevel linear regression analysis, in which effects of age, sex, nutrition and season of birth were adjusted, showing significant difference in ΔLAZ/day between winter and summer with a mean ΔLAZ/day difference of 0.0026 (95%CI 0.0015 to 0.0036; *P* < 0.001). This seasonal difference corresponded to 13% of the average linear growth velocity in 6-month-old infants. A modest effect of nutrition on linear growth was observed with a mean ΔLAZ/day difference of 0.0015 (95%CI 0.0006 to 0.0025; *P* < 0.001) between predominantly formula-fed infants and breastfed infants. In conclusion, we observed that linear growth, but not weight gain, of Japanese infants showed significant seasonality effects represented by increases in summer and decreases in winter.

## Introduction

Growth and growth disorders in early life have been central themes in pediatrics, and there are several theories pertaining to concepts of human growth. The infancy-childhood-puberty (ICP) model^1^ is one of the most popular theories, stemming from the work by Scammon in the 1920s^2^. The model recognizes three different phases of growth including, “Infancy”, known as the period with fastest growth velocity^3^; “Childhood”, characterized by steady growth velocity; and “Puberty”, which is a period of rapid growth with sexual maturity. The greatest growth velocity occurs during the first 6-months in both normal children and children born small-for-gestational age^3,4^. This rapid growth is thought to be influenced by nutritional features characteristic of this development period. Another known factor affecting infant growth is sex. According to the World Health Organization growth standards data, mean increase in length during the first 12 months of life is 25.87 cm in boys and 24.87 cm in girls^3^. A third potential determinant of infant growth is climatic seasons. There have been studies conducted in Ethiopia^5^ and Timor-Leste^6^ reporting the seasonality of infant growth, in which food availability was thought to be the major determinant of the seasonality pattern. To our knowledge, potential effects of the seasons on infant growth in developed countries have not been reported. The scarcity of previous studies may be due, in part, to the inability to collect measurements on child growth in a sufficiently detailed prospective fashion.

Today, mobile devices such as mobile phones, tablet computers and smart watches are in widespread use. There is an abundance of mobile applications (app) aimed at healthcare management. This enables the potential for accessing larger and more diverse pools of participants for research purposes. In this study, the use of a mobile phone app-based data collection strategy gave us a unique opportunity to carefully investigate the role of seasonality on infant growth, a topic that is usually challenging to pursue using traditional methods. We uncover a previously unrecognized link between climatic seasons and linear growth of infants.

## Results

### Descriptive characteristics

**Fig. 1:**
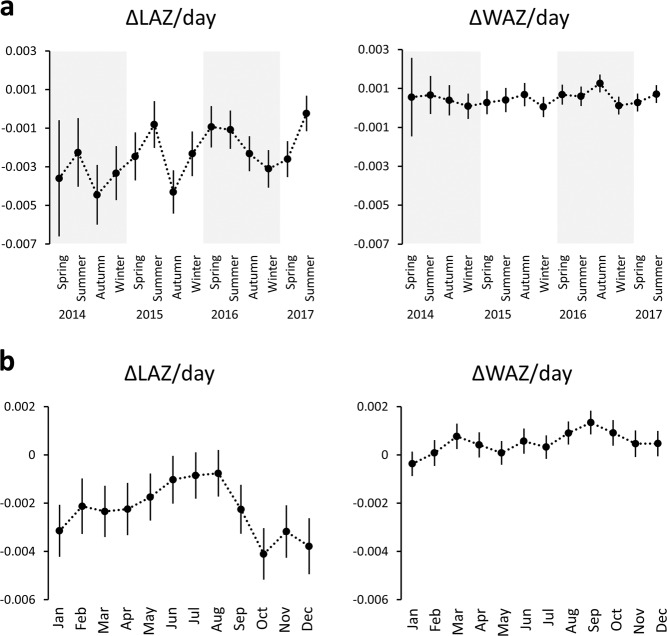
ΔLAZ/day and ΔWAZ/day. ΔLAZ/day and ΔWAZ/day by year-season (**a**) and by month (**b**). Circles indicate means, and lines indicates 95%CI. Note that ΔLAZ/day shows annual fluctuations while ΔWAZ/day does not.

Descriptive characteristics of the study subjects comprising infants 0 to 400 days old are shown in Table 1. Length-for-age and weight-for-age were converted into *z-*scores with age- (in days) and sex-specific references (LAZ and WAZ), and magnitude of change per day (ΔLAZ/day and ΔWAZ/day) was used as the primary outcomes. Out of the 9409 infants, 5870 had at least one ΔLAZ/day (total 20,007 ΔLAZ/day) measurement and 9165 had at least one ΔWAZ/day (total 33,236 ΔWAZ/day) measurement available. A subject with one ΔLAZ/day value meant that at least two LAZ data points were recorded at an interval of 15 to 100 days (see METHODS for details). Means and standard deviations for ΔLAZ/day and ΔWAZ/day were −0.002 ± 0.022 and 0.001 ± 0.014, respectively. To examine the seasonality of linear growth and weight gain, ΔLAZ/day and ΔWAZ/day were aggregated for each year and season, and the fluctuations were visualized (Fig. 1A). ΔLAZ/day showed consistent fluctuations across the years with increases in summer and decreases in winter, while no similar pattern was seen for ΔWAZ/day (Fig. 1A). Aggregation of ΔLAZ/day and ΔWAZ/day by month (across multiple years) revealed that mean ΔLAZ/day was highest in summer (Fig. 1B).

**Table 1:** Characteristics of the subjects.

Variables	
Sex, *N*	
Male	4933
with ≥1 ΔLAZ/day	3104
with ≥1 ΔWAZ/day	4809
Female	4476
with ≥1 ΔLAZ/day	2766
with ≥1 ΔWAZ/day	4356
Season of birth, *N* (%)	
Spring	1936 (20.6)
Summer	2403 (25.5)
Autumn	2758 (29.3)
Winter	2312 (24.6)
Nutritional groups, *N* (%)	
Breastfeeding	4895 (52.0)
Mixed feeding	2434 (25.9)
Formula-dominant feeding	1955 (20.8)
Unknown	125 (1.3)
Age (days) at measurements, median (IQR)^a^	
Length	122 (42–214)
Weight	123 (59–211)
Average number of measurements per child	
Length	4.8
Weight	5.4

*IQR* interquartile range, *LAZ* length-for-age *z-*score; *WAZ* weight-for-age *z*-score.

^a^The values include multiple measurements at different time points per child.

### Multilevel linear regression analysis

In the multilevel linear regression analysis adjusting for age, sex, nutritional group, the period, and season of birth, mean ΔLAZ/day in summer was 0.0026 (95%CI, 0.0015 to 0.0036) higher than that in winter (*P* < 0.001), while those in spring and autumn were not significantly different (Table 2). This summer–winter difference corresponded to 13% of the average linear growth in 6-month-old infants: 6-month-old infants gain 1.3 cm/month on average and the seasonal difference was estimated to be 0.18 cm. The regression analysis also showed an effect of nutrition with a mean ΔLAZ/day of 0.0016 (95%CI, 0.0006 to 0.0025) higher in formula-fed infants than in breastfed infants. Another factor associated with ΔLAZ/day was the period variable, which was likely explained by the difference in growth patterns between the study subjects and the general population used to construct the growth standard in 2000.

**Table 2:** Multilevel linear regression analysis of ΔLAZ/day.

Variable	Estimate (95% CI)	*P-*value
Intercept	−0.0083 (−0.0101 to −0.0064)	<0.001
Age at reference date (days)	0.0000 (0.0000 to 0.0000)	<0.001
Period of measurement		
1	−0.0037 (−0.0050 to −0.0023)	<0.001
2	0.0029 (0.0016 to 0.0042)	<0.001
3	0.0021 (−0.0010 to 0.0033)	<0.001
4	Reference	
Season of reference date		
Spring	0.0008 (−0.0003 to 0.0019)	0.15
Summer	0.0026 (0.0015 to 0.0036)	<0.001
Autumn	0.0010 (−0.0001 to 0.0020)	0.085
Winter	Reference	
Season of birth		
Spring	−0.0006 (−0.0017 to 0.0004)	0.23
Summer	−0.0008 (−0.0018 to 0.0002)	0.13
Autumn	0.0004 (−0.0006 to 0.0014)	0.47
Winter	Reference	
Sex		
Female	0.0004 (−0.0003 to 0.0011)	0.26
Male	Reference	
Nutritional group		
Formula-dominant	0.0015 (0.0006 to 0.0024)	0.001
Mixed	−0.0005 (−0.0013 to 0.0004)	0.27
Breast	Reference	

## Discussion

Using a mobile phone app-based data collection strategy, we provided a first account of empirical evidence showing a seasonality effect on linear growth in human infants. We analyzed a unique dataset assembled based on voluntary measurement entries by app users for the purpose of tracking the growth of their child. As with any data collection approach, we assume there to be a certain degree of measurement error, but likely random in nature. We might expect generally high-quality data, as the use of this app was not compulsory, but instead motivated by self-interests and care for their own infant.

Enhanced growth from spring to summer has been reported among toddlers and school children in small-scale observational studies^6–11^, although the mechanisms are unknown. Surprising of this current study was that the seasonal variation in linear growth was also observed in infants, even though the ICP model indicates that the determinants of growth are quite different between infancy and childhood^1^. This previously unrecognized consistency provides new insight into the mechanisms of the seasonal variation. In low-income countries, higher growth velocity has been observed in seasons with better food availability^5,6^. This mechanism may not apply to our population, as nutrition intake seemed to remain constant throughout the years in our study since weight gain did not show seasonality. The second candidate mechanism is hormonal control of growth via change in growth hormone (GH) secretion levels. However, this mechanism is also unlikely because infant growth is known to be mostly GH-independent^1^, and the seasonality of linear growth has been observed in GH-deficient children^12^. The third candidate mechanism is related to vitamin D. Serum levels of vitamin D, of which deficiency causes growth restriction^13^, shows variation by season, with higher levels in summer and lower levels in winter^14^. As data on vitamin D sufficiency is beyond the scope of the current analysis, we were unable to evaluate the validity of this hypothesized mechanism. The amplitude of the seasonal variation of linear growth may not be large enough to have direct implications on clinical practice. However, understanding the patterns across seasons can be of public health relevance to indicate potential concerns related to, for example, population-level deficiencies in vitamin D. At present, since data is not sufficient to speculate on the reasons for seasonal variation, additional studies will be important to help clarify the implications of these findings.

Determinants of infant weight gain has been studied extensively due to its association with risks for adulthood cardiovascular disorders^15^. In contrast, only few have studied determinants of infant linear growth^12^. In our study, we observed a marked effect of seasonality on infant growth, as well as an effect of nutrition. The difference in linear growth velocity, although significant, was small, and may have been detectable due to our large sample size. Such a pattern would not be recognizable in routine health checkups particularly considering common errors in length measurements that are typically 0.3 to 0.5 cm. In our study, we also observed a modest effect of nutrition on linear growth. Previous reports on the effect of nutrition in developed countries have been inconclusive with studies showing both positive and negative associations^15,16^.

The number of births in Japan during the study period was about 3.5 million, and thus the present study covered ~0.3% of the general population. The proportions by sex and breastfeeding practices in this study were comparable to the general Japanese population (52% vs. 51% and 51% vs. 52%, respectively; https://www.mhlw.go.jp/toukei/list/83-1c.html). Considering the nationwide coverage of participants and representativeness of these two major determinants of infant growth compared to national distributions, we interpret our results of the effect of season on linear growth to be generalizable to the Japanese population. Understanding the consistency of finding across different regions of the world will be of interest for future studies.

This study has certain limitations. First, we did not have access to biochemical data (e.g., GH, IGF1, vitamin D, etc.), which could have been used to help elucidate potential mechanisms for the effect of seasonality on linear growth. Second, we used self-reported data that may not have been based on standard methods of measurement across individuals. This may have led to attenuation bias due to the non-differential misclassification of measurements. Third, data on residential area of the app users were not accessible, precluding the ability to test the effect of latitude coordinates that have been implicated in studies of seasonality on height growth in children^17^. Nevertheless, more than 80% of Japanese live in areas with latitude coordinate between 33 and 37 degrees, and thus the effect, if any, is expected to be minimal. Fourth, we did not analyze weight-for-height, an estimate of ponderal growth, due to the lack of age-specific standardized data. We acknowledge that WAZ is a composite indicator of growth of length and weight. The effect of seasons on ponderal growth of infants examined using weight-for-height measures would be an important area for future research.

In conclusion, we conducted a mobile phone app-based analysis of growth of Japanese infants, and found that the ΔLAZ/day in summer was significantly higher than winter. Our findings shed light on a previously unrecognized role of seasons on infant linear growth, which is still considered a mystery of modern pediatrics.

## Methods

### Study population

The population for this study was assembled based on use of a free mobile phone app called, Papatto Ikuji. This free app was developed by First Ascent Inc., which was created for the purpose of childcare support. The app can be used anonymously on Android and iOS platforms. Caregivers voluntarily entered their child’s records and schedule pertaining to daily life activities and occurrences, including breastfeeding, formula-feeding, defecation, bath, sleep, as well as measurement data. Papatto Ikuji is one of the most popular apps used by caregivers of infants in Japan; it was installed by 73,496 users in 2016 that corresponded to 7.5% of newborns in Japan that year.

During the period of January 2014 to October 2017, Papatto Ikuji was installed by 234,781 caregivers, and used for 295,957 children. Of these 295,957 children, one or more length and weight measurements were recorded for 15,948 children and 22,067 children, respectively. When limited to measurements up to 400 days of age, 6963 infants and 12,070 infants had two or more length and weight measurements, respectively. Infants aged 0 to 400 days with two or more measurements recorded at intervals of 15 to 100 days were included. This lower bound of the measurement interval was set in order to maximize the number of biologically meaningful measurements considering that small differences observed between those taken within 2 weeks of each other may be within the range of expected measurement error. The upper bound was set to approximate the length of an average season. A total of 9558 infants fulfilled the criteria, including 5957 with length data and weight data, 3397 with weight data only, and 204 with length data only. Medians (interquartile range) for the number of measurements were 4 (2 to 7) for length and 4 (2 to 8) for weight. It was assumed that the majority of user-entered data were based on measurements taken at clinics and hospitals during routine health checkups and vaccinations, but a small portion may have been performed at home by a parent. Measurements taken at clinics or hospitals are based on crown-heel lengths measured using an infantometer with the child lying supine, and weight was measured using a digital weighing machine.

This research was considered as secondary use of data and was approved by the ethics committee of the National Center for Child Health and Development. Requirement for written informed consent was waived. All users were given the opportunity to opt out by notification through the app. Information on unique identifiers were removed to create an anonymized dataset for the study.

### Data collection

In the present study, the following data were collected and analyzed: sex, birthdate, measurement data (length, weight, date of measurements), date of measurements, and numbers of times of breast- and formula-feeding. Measurement data were converted into *z-*scores with use of Japanese age- (in days) and sex-specific references (LAZ and WAZ), using a Microsoft Excel spreadsheet app distributed by the Japan Society for Pediatric Endocrinology (http://jspe.umin.jp/medical/chart_dl.html). LAZ or WAZ data less than −3.0 or more than +3.0 were excluded. This procedure excluded 635 out of 29,306 LAZ values (2.2%) and 604 out of 51,671 WAZ values (1.2%). Inspection of the excluded data showed that exclusion was chiefly due to errors in input such as entering length and weight in reverse. The full data are available in figshare (https://figshare.com/).

We calculated the ΔLAZ/day and ΔWAZ/day for a given date (referred to as a reference date; the midpoint of the dates of two measurements) using the following formula:

$${\Delta} {\mathrm{LAZ}}/{\mathrm{day}} = \frac{{\left( {{\mathrm{LAZ}}_{{\mathrm{T}}2} - {\mathrm{LAZ}}_{{\mathrm{T}}1}} \right)}}{{{\mathrm{T}}2 - {\mathrm{T}}1}},$$

$${\Delta} {\mathrm{WAZ}}/{\mathrm{day}} = \frac{{\left( {{\mathrm{WAZ}}_{{\mathrm{T}}2} - {\mathrm{WAZ}}_{{\mathrm{T}}1}} \right)}}{{{\mathrm{T}}2 - {\mathrm{T}}1}},$$

where LAZ_T1_ and LAZ_T2_ were the values of LAZ for two consecutive measurements, and T1 and T2 were the dates of the two corresponding measurements.

In order to assess seasonal difference of growth velocity, we assigned seasons or months for each ΔLAZ/day (or ΔWAZ/day) based on the season of the reference date. Seasons were assigned as winter (December to February), spring (March to May), summer (June to August), and autumn (September to November) based on classification standards of the Japan Meteorological Agency. For example, if two consecutive time points were July 1 and August 4, the reference date is July 18, and the ΔLAZ/day and ΔWAZ/day data were allocated to summer.

We categorized nutritional groups based on a breastfeeding index, which was calculated by (number of times of breastfeeding)/(number of times of breastfeeding + number of times of formula). Three groups were defined: breastfeeding group (index 0.90 or more), mixed feeding (index 0.50 to 0.89), and formula-dominant feeding (index < 0.50).

### Statistical analysis

We reported the descriptive characteristics of the cohort as counts and percentages for categorical variables, median and interquartile range for non-normally distributed variables, and mean and standard deviation for normally distributed variables, as appropriate. ΔLAZ/day and ΔWAZ/day for each year and season were presented along with means and 95% confidence intervals (CI). Data were also aggregated by combining data from the same months across the 4 years.

**Fig. 2:**
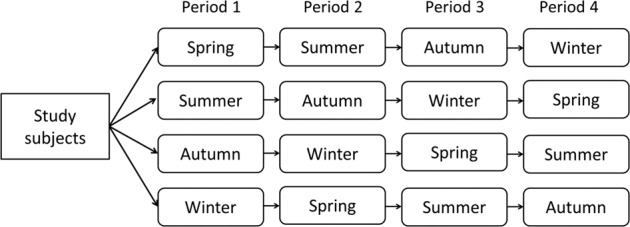
Relationships between the season of birth and the time sequence. Period 1 was defined as the season of birth. Period *N* of a measurement was defined as the number of seasons subsequent to the season of birth within which the measurement was made. The multilevel linear regression analysis was conducted as a crossover randomized control trial-like method with fitting the time sequence as a random effect.

We conducted multilevel linear regression analysis to evaluate the association between ΔLAZ/day and seasonality while controlling for correlations between repeated measurements of the same individual. To decrease the effect of systematic error due to repeated measurements, we allowed only one ΔLAZ/day per season for this analysis (Fig. 2). Mean ΔLAZ/day was calculated if there was more than one reference date in each season. The regression model included age at reference date (averaged if multiple ΔLAZ/day were available for a season), season of reference date, season of birth, sex, nutritional group, and time sequence. The time sequence (referred to as Period 1 to Period 4) was a number calculated from the season of birth to the season of reference date (Fig. 2). For example, the Period for the timing of a measurement is 2 if an infant was born in summer and the reference date was autumn. Since age of the study population was restricted to age 400 days or less, up to 4 periods are applicable to each infant (Period 1 to 4) corresponding to the interval across 4 possible seasons specific to the timing of their ΔLAZ/day measure. The time sequence was fitted as a random effect in this model. The models were fitted using maximum likelihood estimation. We used the statistical software package SAS 9.4 (SAS Institute, Cary, NC) for all data analyses.

### Reporting summary

Further information on research design is available in the Nature Research Reporting Summary linked to this article.

## Supplementary information

Reporting Summary

## Data Availability

All raw measurement data files are available from Figshare (https://figshare.com/articles/dataset/Evaluating_the_Seasonality_of_Growth_in_Infants_Using_a_Mobile_Phone_Application/11341163).
