# Supplementary material for: Evaluating the seasonality of growth in infants using a mobile phone application
Source: NPJ Digit Med. 2020 Oct 21;3:138. doi: 10.1038/s41746-020-00345-9 (PMC7578091; doi:10.1038/s41746-020-00345-9)
Supplement: Supplementary file 1 — Reporting Summary [file 41746_2020_345_MOESM1_ESM.pdf]

nature research

Corresponding author(s): Satoshi Narumi, M.D., Ph.D.

Last updated by author(s): Jun 10, 2020

Reporting Summary

Nature Research wishes to improve the reproducibility of the work that we publish. This form provides structure for consistency and transparency in reporting. For further information on Nature Research policies, see our Editorial Policies and the Editorial Policy Checklist.

Statistics

For all statistical analyses, confirm that the following items are present in the figure legend, table legend, main text, or Methods section.

n/a	Confirmed	
☐	☒	The exact sample size (*n*) for each experimental group/condition, given as a discrete number and unit of measurement
☐	☒	A statement on whether measurements were taken from distinct samples or whether the same sample was measured repeatedly
☐	☒	The statistical test(s) used AND whether they are one- or two-sided<br>*Only common tests should be described solely by name; describe more complex techniques in the Methods section.*
☐	☒	A description of all covariates tested
☐	☒	A description of any assumptions or corrections, such as tests of normality and adjustment for multiple comparisons
☐	☒	A full description of the statistical parameters including central tendency (e.g. means) or other basic estimates (e.g. regression coefficient) AND variation (e.g. standard deviation) or associated estimates of uncertainty (e.g. confidence intervals)
☐	☒	For null hypothesis testing, the test statistic (e.g. *F*, *t*, *r*) with confidence intervals, effect sizes, degrees of freedom and *P* value noted<br>*Give P values as exact values whenever suitable.*
☒	☐	For Bayesian analysis, information on the choice of priors and Markov chain Monte Carlo settings
☐	☒	For hierarchical and complex designs, identification of the appropriate level for tests and full reporting of outcomes
☐	☒	Estimates of effect sizes (e.g. Cohen's *d*, Pearson's *r*), indicating how they were calculated

Our web collection on statistics for biologists contains articles on many of the points above.

Software and code

Policy information about availability of computer code

Data collection	Microsoft Excel 2013
Data analysis	Microsoft Excel 2013

For manuscripts utilizing custom algorithms or software that are central to the research but not yet described in published literature, software must be made available to editors and reviewers. We strongly encourage code deposition in a community repository (e.g. GitHub). See the Nature Research guidelines for submitting code & software for further information.

Data

Policy information about availability of data

All manuscripts must include a data availability statement. This statement should provide the following information, where applicable:

- Accession codes, unique identifiers, or web links for publicly available datasets
- A list of figures that have associated raw data
- A description of any restrictions on data availability

The full data are available in figshare (https://figshare.com/). The data can be found by searching with "Evaluating the Seasonality of Growth in Infants Using a Mobile Phone Application"

Field-specific reporting

Please select the one below that is the best fit for your research. If you are not sure, read the appropriate sections before making your selection.

☒ Life sciences ☐ Behavioural & social sciences ☐ Ecological, evolutionary & environmental sciences

For a reference copy of the document with all sections, see nature.com/documents/nr-reporting-summary-flat.pdf

Life sciences study design

All studies must disclose on these points even when the disclosure is negative.

Sample size	In this study, 9,409 Japanese infants were enrolled in the study. This sample size is more than 10 times larger than any previous studies conducted for the similar purpose (Dalskov SM et al., Pediatr Research 2016; sample size N=760).
Data exclusions	The measurement data less than -3.0 or more than +3.0 were excluded. This procedure excluded 635 out of 29,306 LAZ values (2.2%) and 604 out of 51,671 WAZ values (1.2%).
Replication	The similar seasonality was confirmed in three consecutive years.
Randomization	Not applicable to our study.
Blinding	Not applicable to our study.

Reporting for specific materials, systems and methods

We require information from authors about some types of materials, experimental systems and methods used in many studies. Here, indicate whether each material, system or method listed is relevant to your study. If you are not sure if a list item applies to your research, read the appropriate section before selecting a response.

Materials & experimental systems

n/a	Involved in the study	
☒	☐	Antibodies
☒	☐	Eukaryotic cell lines
☒	☐	Palaeontology and archaeology
☒	☐	Animals and other organisms
☐	☒	Human research participants
☒	☐	Clinical data
☒	☐	Dual use research of concern

Methods

n/a	Involved in the study	
☒	☐	ChIP-seq
☒	☐	Flow cytometry
☒	☐	MRI-based neuroimaging

Human research participants

Policy information about studies involving human research participants

Population characteristics	Japanese infants, corresponding to about 0.3% of the general Japanese infants.
Recruitment	The recruitment was performed via a smartphone app "Papatto Ikuji".
Ethics oversight	The Ethics Committee of National Center for Child Health and Development approved this study.

Note that full information on the approval of the study protocol must also be provided in the manuscript.
